# Frequency Detection for String Instruments Using 1D-2D Non-Contact Mode Triboelectric Sensors

**DOI:** 10.3390/mi15091079

**Published:** 2024-08-26

**Authors:** Inkyum Kim, Hyunwoo Cho, Daewon Kim

**Affiliations:** 1 Department of Electronics and Information Convergence Engineering, Institute for Wearable Convergence Electronics, Kyung Hee University, 1732 Deogyeong-daero, Giheung-gu, Yongin 17104, Republic of Korea; 2 Department of Electronic Engineering, Institute for Wearable Convergence Electronics, Kyung Hee University, 1732 Deogyeong-daero, Giheung-gu, Yongin 17104, Republic of Korea; 3 Center for Brain Technology, Korea Institute of Science and Technology, 5, Hwarang-ro 14-gil, Seongbuk-gu, Seoul 02792, Republic of Korea

**Keywords:** triboelectric nanogenerators, one-dimensional, string vibration, frequency sensing, string instrument tuning, self-sustainable sensor

## Abstract

The proliferation of small electronic devices has significantly increased the demand for self-powered sensors. This study introduces a triboelectric frequency sensor (TFS) that combines the frequency-responsive characteristics of triboelectric nanogenerators with a simple one-dimensional structure for sustainable vibration measurement. This sensor is specifically designed to aid in the tuning of string instruments, capable of detecting frequency responses up to 330 Hz generated by string vibrations. Structural optimization was achieved by setting a non-contact mode with a gap distance of 3 mm and utilizing perfluoroalkoxy alkane (PFA) as the contact dielectric material. The TFS exhibits dynamic response characteristics by varying the vibrating frequency and the tension of the string, facilitated by a custom-built testing setup. Frequency data captured by the sensor can be visualized on a monitor through the integration of a microcontroller unit (MCU) and dedicated coding. The practical applicability and effectiveness of this sensor in real-world scenarios are demonstrated experimentally. This innovation represents a significant step forward in the development of self-sustaining sensing technologies for precision instrument tuning.

## 1. Introduction

The advent of the fourth industrial revolution has catalyzed a significant increase in the number of Internet of Things (IoT) devices [1]. This surge necessitates innovations in energy management, particularly for sensors, which are integral to IoT systems. To address the escalating energy demands, there is a growing emphasis on sensors operating in a self-powered manner [2,3,4]. Energy harvesting technology offers a promising solution by enabling sensors to operate independently of conventional energy sources [5]. This approach not only reduces reliance on external power supplies but also enhances the sustainability and efficiency of IoT systems. By converting ambient energy from the environment into usable electrical power, sensors can continuously function without the need for continuous external energy inputs. The integration of triboelectric nanogenerators (TENGs) represents a pioneering advancement in this field [6,7,8,9]. These devices exploit the natural interactions between materials to generate electricity from mechanical movements, such as vibrations or rotations, commonly encountered in industrial applications [10]. The potential to harness this technology for self-sustaining sensors could profoundly impact IoT deployment, rendering devices more versatile and environmentally friendly.

To effectively harvest energy from dynamic conditions, a TENG emerges as a suitable alternative, thanks to its ability to respond adaptively to object movements [11,12]. TENGs operate by generating Maxwell’s displacement current through the combined mechanisms of contact electrification and electrostatic induction [13]. This technology leverages the natural interactions between different materials, which come into contact and then separate, generating electrical charge through displacement. The wide range of material options suitable for constructing TENGs, along with their straightforward operating principles, further underscores their utility for self-powered sensors [14]. These characteristics render TENGs particularly appealing for integration into various applications, where they provide a reliable power source by converting mechanical energy from environmental movements into electrical energy. This conversion process not only enhances the functionality of sensors in remote or inaccessible locations but also contributes to the sustainability and autonomy of monitoring systems across different sectors [15,16,17,18]. Furthermore, the scalability and versatility of TENG technology allow for its implementation in a diverse array of settings, from industrial machinery monitoring to environmental sensing, investigating its potential as a transformative component in the development of self-sustaining IoT systems [19,20].

For vibration frequency sensing, TENG devices offer advantages due to their compact and lightweight design, rendering them suitable for constrained spaces and small electronic applications [21]. Advanced designs, such as those incorporating quasi-zero stiffness structures or magnetically floating systems, provide high sensitivity and precision in detecting vibrations, often with errors below 0.5% [22]. Additionally, TENG sensors can detect a wide range of frequencies, from low to high (e.g., 2–13,000 Hz), enabling them to be versatile for various applications, including sound detection [23,24,25,26]. However, a current limitation of TENG-based frequency sensing technologies is material wear, especially in contact elements, which can affect long-term reliability. The non-contact mechanism addresses the issue of ensuring that vibrations are not altered during measurement, thereby preserving data integrity. This approach also simplifies setup and minimizes the risk of sensor interference with the object being measured, enhancing both the durability and consistency of measurements.

Research has also extended to string-based electronics, which are being explored for their potential in wearable applications when integrated into fabrics [27,28,29,30,31,32]. String-based one-dimensional (1D) devices are particularly suitable for vibration sensing applications due to their simple structure and easily quantifiable characteristics [33,34]. Unlike prior research that predominantly focused on two-dimensional (2D) structures or rigidly mounted 1D structures [35,36,37,38], these flexible and string-like sensors offer an efficient approach to capturing and analyzing mechanical movements. This ensures they are ideal for incorporation into textiles requiring flexibility and durability. The versatility of string-based sensors allows for enhanced integration into everyday objects and garments, opening new avenues for interactive and responsive technology in personal electronics and healthcare monitoring. This gap in the research highlights a significant opportunity for further exploration of string-based 1D sensors in wearable technologies.

This paper introduces a triboelectric frequency sensor (TFS), an attachable device designed for sensing string vibrations. The TFS measures the frequency of string vibrations by optimizing the materials and structural factors of the device through a vibration-injecting setup. Tribo-negative materials were used for the CDL of the TFS to enhance the triboelectric output in combination with nylon wire, which exhibits highly tribo-positive characteristics. Al was chosen for the EL due to its conductive properties and availability, thereby increasing the sensor’s compatibility. The durability of the TFS has been rigorously tested across numerous operating cycles to ensure its reliability. In the same experimental setup, variations in frequency are achieved by adjusting the speed of the motor and the tension of the string. The fast Fourier transform (FFT) is employed to visualize the frequency response of the electrical outputs, providing a clear depiction of the sensor’s performance under different conditions. Comparative tests with a commercial tuner demonstrate the applicability of TFS to real-world music applications, such as adjusting the scale of a ukulele by manipulating the tuning pegs. A microcontroller unit (MCU) is utilized to capture electrical output signals and convert these into a frequency response, which is subsequently displayed on a monitor using Python coding. Moreover, the adaptability of this system extends beyond musical applications; it also offers potential uses in safety-related applications where precise vibration monitoring is essential. By enhancing the accuracy and usability of vibration sensing in string instruments and potentially other contexts, the TFS represents a significant advancement in sensor technology.

## 2. Design of the Sensing Device

**Figure 1:**
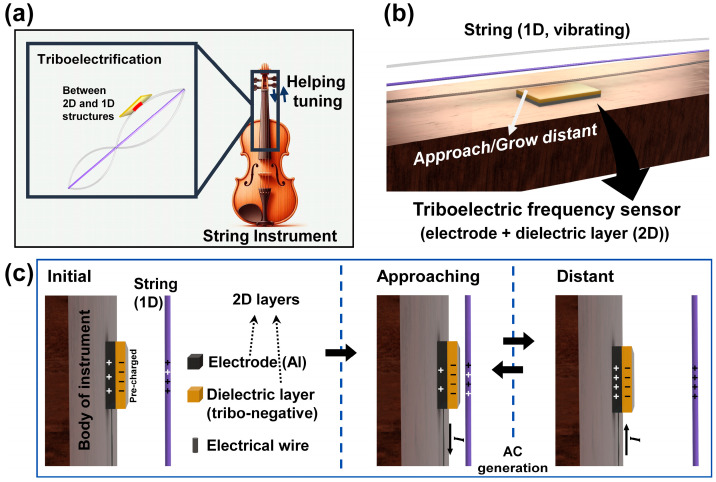
Structure and schematic of the TFS. (**a**) Target location for applying TFS to a string instrument. (**b**) Simple schematic for detecting string vibration with the TFS. (**c**) Structure and operating principle of the TFS.

The innovative approach to frequency detection in string instruments leverages a 1D triboelectric sensor, as illustrated in Figure 1a. This design capitalizes on the non-contact sensing capability of the 1D string, which allows for the measurement of vibration frequencies without the need for precise alignment between the string and the (non-)contact dielectric layer (CDL). This feature significantly simplifies the setup and reduces potential errors due to misalignment, which can be critical in traditional contact-based sensing methods.

The simplified schematic of the sensor device, shown in Figure 1b, provides insight into its construction and operational principles. The device comprises an electrode layer (EL) and a CDL. The EL, indicated in brown-gray, is fabricated from aluminum, known for its excellent electrical conductivity under various operating conditions. The CDL, represented in beige, is composed of perfluoroalkoxy alkane (PFA), a fluoropolymer known for its high chemical resistance, low coefficient of friction, and excellent dielectric properties. The selection of PFA for the CDL is strategic, aiming to enhance the sensitivity and efficiency of the triboelectric effect in generating electrical signals.

The structural design of the sensor is meticulously planned to maintain a specific height between the string and the CDL. This configuration is critical as it defines the optimal distance required for efficient triboelectric interactions. The non-contact mode of the sensor allows it to detect vibrations induced in the string by external forces without physically altering the string’s natural vibrational characteristics. This non-intrusive measurement approach ensures that the integrity and quality of the sound produced by the string are preserved.

Upon the application of an external force, the string vibrates and these vibrations are detected by the triboelectric sensor, which then generates an alternating current (AC) signal. The amplitude and frequency of this AC signal correlate directly with the vibration characteristics of the string. By analyzing these electrical signals, detailed information about the vibration frequency and intensity can be extracted. These data are invaluable for applications in acoustics and performance monitoring, as they provide a non-invasive means of assessing and tuning the instruments.

## 3. Operating Modes and Principle of the Device

TENG operates based on a fundamental mechanism involving the generation of current through the dynamic interaction between the string and the CDL. Figure 1c illustrates this process, where initially, the string and the CDL are physically separated. As these components engage in repeated cycles of approaching and distant states, triboelectric charges accumulate on their surfaces; positive charges accumulate on the string, while negative charges collect on the CDL. This charge separation is a result of the differing electron affinities of the materials, which induce charge transfer when they come into contact.

During these cycles, as the distance between the string and the CDL fluctuates, an electric field is established. This field prompts positive charges in the EL to migrate in an attempt to neutralize the system’s overall charge imbalance. This movement of charges through the electrical circuitry connected to the EL generates a measurable current. Specifically, the migration of positive charges in response to the approaching state between the string and CDL leads to the production of a direct current (DC). However, as the string and CDL grow distant, the polarity of the charges is reversed, resulting in a reverse current. The continuous back-and-forth motion of the string thus induces an AC, driven by the changing electric field and the resultant charge flow.

In contrast to the non-contact mode illustrated in Figure 1c, the contact mode of the TFS, as depicted in Figure S1, involves direct physical contact between the string and the CDL. This contact mode enhances the efficiency of charge transfer, leading to a more substantial flow of charges and consequently a higher AC output compared to the non-contact mode. The increased surface interaction in the contact mode facilitates more robust charge induction, which is crucial for applications requiring stronger signal output.

Further analysis of the non-contact mode of the TFS was conducted using the finite element method (FEM) in COMSOL Multiphysics 5.0. This simulation provided a detailed charge distribution profile, allowing for a comprehensive understanding of the device’s electrostatic behavior under varying conditions. As depicted in Figure S2a–c, the study examined the electrical potential values at different distances between the 1D string and the 2D CDL: 2.95 mm, 1.5 mm, and 0.05 mm. The corresponding potential differences were recorded as 15.73 V, 11.68 V, and 3.98 V, respectively. These findings emphasize the relationship between the distance in the non-contact mode and the generated electrical potential, demonstrating that as the gap narrows, the potential decreases due to the reduced area for charge induction.

This analysis confirms that the output in the non-contact mode is primarily driven by electrostatic induction, highlighting the importance of precise control over the gap distance to optimize the sensor’s performance. The ability to operate in both contact and non-contact modes offers flexibility in application, satisfying different sensing requirements and environmental conditions. The data obtained from these analyses provide critical insights into the design and optimization of TENG devices for various practical applications.

## 4. Experimental Results and Discussion

### 4.1. Measurement of the Electrical Outputs from the TENG and Coding for the Frequency Sensing Application

To collect the electrical output data, a system electrometer (Keithley Model 6514, Solon, OH, USA) was employed, interfaced with a multi-channel DAQ system (NI PCI-6220, Austin, TX, USA). This setup facilitated the precise measurement and recording of electrical signals generated by the TENG.

Real-time visualization of the signals from the analog channel of the Arduino Nano was achieved through a combination of Arduino and Python coding. The Python environment was enhanced with several libraries, including PyQt5 (version 5.15.10) for graphical user interface development, matplotlib for plotting, and datetime for managing time-series data. The test setup utilized a string with a diameter of 0.7 mm, matching the thickness of the test string used in the ukulele, to ensure consistency in the experimental conditions.

### 4.2. Setup for Measurement and Basic Electrical Output Characteristics

**Figure 2:**
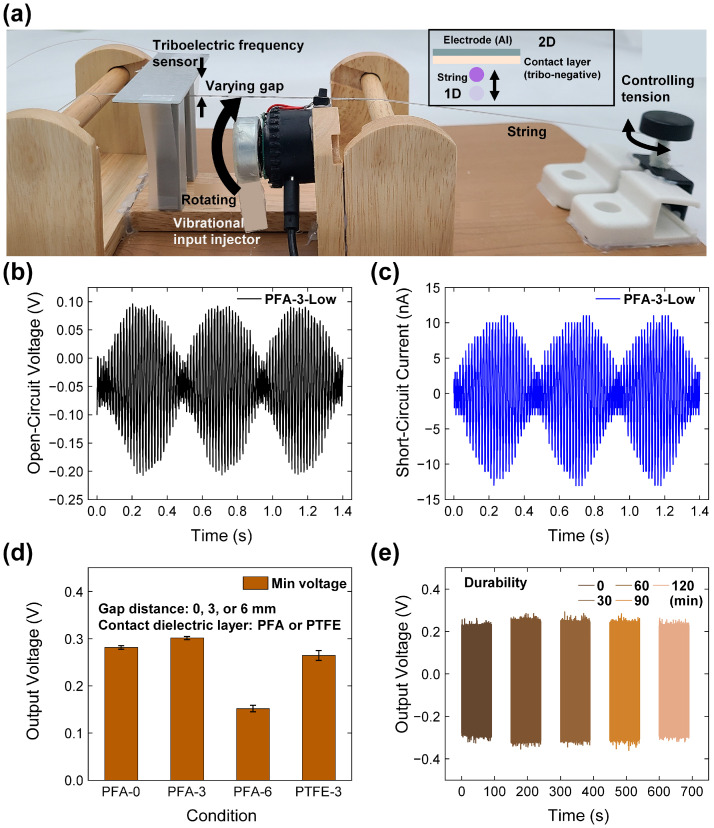
(**a**) Structure for the test setup for injecting vibration to string and demonstrating the TFS. Output signals for (**b**) open-circuit voltage and (**c**) short-circuit current. (**d**) Output voltage comparison with changing gap distance and contact dielectric material. (**e**) Durability test result for 416,160 cycles.

Figure 2a presents a schematic of the test conditions for introducing vibration input to the TFS. As depicted in the inset of Figure 2a, a rotating film interacts with the wire, driven by the direct current (DC) motor. Upon activating the switch, the speed of the DC motor can be adjusted across three distinct levels.

The TFS itself is securely attached to a glass substrate, with an electrical wire connected to the exposed area of the electrode. To facilitate varying operational conditions, the gap distance between the downward-facing TFS and the string is adjustable via the use of different heights of 3D-printed pillars crafted from acrylonitrile butadiene styrene (ABS) filament positioned beneath the TFS. Additionally, the tension of the string can be modified by rotating the black lever located at the right end of the setup. This arrangement allows for precise control over the mechanical conditions impacting the TFS, ensuring that the sensor’s response to varying vibrations can be accurately analyzed. The ability to adjust the gap distance and string tension provides valuable insights into how these variables influence the electrical output and overall performance of the TFS.

Figure 2b,c illustrate the output characteristics of the TFS in terms of open-circuit voltage (*V*_OC_) and short-circuit current (*I*_SC_), which were obtained through vibration inputs facilitated by the DC motor. During the tests, a low-frequency periodic signal with a duration of 0.47 s was observed. Additionally, high-frequency peaks corresponding to the vibration frequency of the string were noted, with periods clocking in at 0.017 s. Analysis of these signals revealed the highest peak-to-peak value of the *V*_OC_ to be 0.298 V, and the average peak value of the *I*_SC_ reached 12 nA. These measurements reflect the sensor’s ability to capture and convert mechanical vibrations into electrical signals with considerable precision. The distinct frequencies captured in the output signals provide valuable insights into the dynamic response of the TFS to varying vibrational inputs. The capability to detect both low and high-frequency vibrations underscores the suitability of TFS for applications requiring sensitive and accurate vibration monitoring. This level of detail in frequency response highlights the sensor’s potential for nuanced control and application in fields where precise vibration detection is crucial.

Figure 2d illustrates the output response variations caused by different gap distances between the CDL and the string. The gap distances tested were 0 mm, 3 mm, and 6 mm, labeled as PFA-0, PFA-3, and PFA-6, respectively. Among these, the highest voltage output of 0.301 V was observed in PFA-3, which is positioned just before the point of contact. This output represents a 98.4% increase compared to PFA-6 and a 14% increase compared to PTFE-3. This optimal gap allows for significant vibrational freedom, thereby maximizing electrical output. In contrast, the contact state with the PFA-0 configuration resulted in lower electrical output than that from PFA-3, as the reduced movable range of the string in vibration mode hindered the generation of electrical energy. Additionally, a notable frequency reduction was observed; the frequency decreased by 1.21%, dropping from 57.8 Hz in the non-contact mode to 57.1 Hz in the contact mode. This decrease underscores the detrimental effect of smaller gap distances on the system’s performance. These findings highlight the importance of maintaining a gap distance of over 3 mm, ensuring that the string has enough room to vibrate without contact, thereby optimizing the sensor’s electrical output and operational frequency. This strategic setup enhances the sensitivity and reliability of the TFS in capturing and converting mechanical vibrations into electrical signals. The optimal gap distance of 3 mm was maintained while experimenting with different materials to further enhance the sensor’s performance.

The material initially used, PFA, was compared with polytetrafluoroethylene (PTFE), which is known for its highly tribo-negative properties. Despite the strong tribo-negative characteristics of PTFE, it was observed that PFA exhibited more pronounced tribo-negative characteristics than PTFE [39]. This difference in material properties led to a 14.0% increase in the electrical output when using PFA, as demonstrated in the results shown in Figure 2d. Given these findings, the use of PFA in the PFA-3 setup was retained for subsequent experiments. The selection of PFA not only aligns with the observed data but also supports the broader goal of optimizing the triboelectric frequency sensor’s efficiency and reliability in practical applications.

The durability of the TFS was rigorously tested to assess its stability over extended use. Figure 2e illustrates the results of these durability tests, where the sensor was subjected to 416,160 cycles of vibrational input over a period of 120 min. Following this intensive testing regime, the output voltage of the TFS exhibited a minimal decrease, with only a 1% reduction observed. This result underscores the robustness of the TFS, confirming its potential for long-term applications as a durable and stable vibrational sensor. The slight decrease in output after numerous cycles demonstrates the sensor’s ability to maintain a high level of performance despite continuous stress, establishing it as ideal for applications where reliability is critical. The ability of the TFS to withstand such a high number of cycles without significant degradation is indicative of its excellent material properties and design efficacy.

### 4.3. Electrical Outputs Varying Motor Speed and String Tension with Device Optimization

Using the switch depicted in Figure 2a, the rotation speed of the DC motor was adjusted across three distinct levels. To adjust the motor’s rotating speed, the voltage applied to the motor was set to 0.82 V, 1.08 V, and 1.46 V for low, medium, and high speeds, respectively. This alteration in motor speed enabled the examination of the frequency-sensing characteristics of TFS under varying input vibration frequencies.

**Figure 3:**
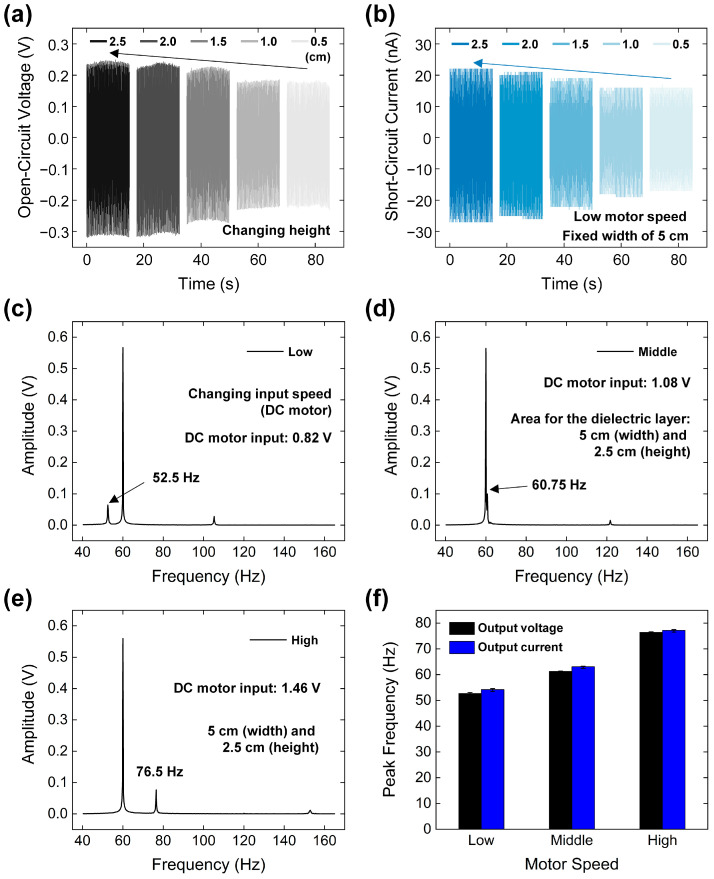
Optimization of the TFS area with (**a**) output voltage and (**b**) output current. FFT spectra of the output voltage at motor input speeds of (**c**) low, (**d**) medium, and (**e**) high. (**f**) Summary of frequency-output results with varying motor input speeds.

The TFS area was optimized for sensing the string’s vibration, as shown by the electrical outputs in Figure 3a,b. The height of the TFS was varied from 0.5 cm to 2.5 cm in 0.5 cm increments, while the width was fixed at 7.5 cm for the EL and 5 cm for the CDL. The 2.5 cm configuration showed a 40.2% increase in output voltage and a 48.5% increase in output current compared to the 0.5 cm configuration. The output voltage and current increased with the larger effective sensing area between the TFS’s 2D layers and the string [40]. When the TFS height approached the string’s vibration length, the rate of increase in both outputs decreased above 2 cm. Despite the continued increase in electrical outputs beyond a 2 cm height, a height of 2.5 cm was chosen for further testing based on the first sample’s results after the slope decrease.

At the lowest speed setting, the peak frequency was identified using the FFT analysis. It is important to note that a peak commonly associated with measuring equipment noise at 60 Hz was removed to ensure accuracy in the results. Subsequently, the peaks were observed at a frequency of 52.5 Hz, as shown in Figure 3c.

For the mid and high-speed settings, the FFT analysis revealed peaks at higher frequencies. Specifically, peaks appeared at 60.75 Hz and 76.5 Hz for the mid and high settings, respectively, as illustrated in Figure 3d,e. These results highlight the capability of TFS to reliably detect changes in vibration frequency induced by variations in motor speed, demonstrating its sensitivity and effectiveness in dynamic environments.

The organized results displaying the output current are presented in Figure 3f. These results illustrate a consistent trend where both the output voltage and output current exhibit a gradual increase in response to the varying input frequencies. This trend demonstrates the sensor’s ability to maintain a uniform response across different types of electrical outputs under changing dynamic conditions. Further insights into the frequency response of the output current are provided by the FFT spectra, shown in Figure S3, which display frequency peaks at 54.24 Hz (low speed), 63 Hz (middle speed), and 77.25 Hz (high speed).

As indicated in Figure S3, the frequency peaks of the output current differ from those of the output voltage. The open-circuit voltage exhibits square wave characteristics, while the short-circuit current shows impulse-like characteristics with two polarities. Consequently, accurate frequency detection of the output current is more challenging compared to the output voltage, given the electrometer’s resolution of 1200 readings per second. This can lead to greater fluctuation in peak frequency detection with the output current.

**Figure 4:**
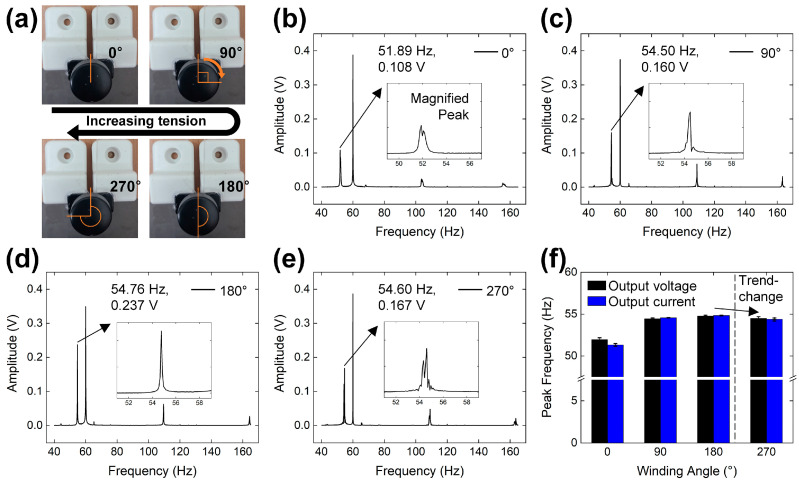
(**a**) Digital camera images of string tension control with the winding angle of the lever. FFT spectra of output voltage with winding angles of (**b**) 0, (**c**) 90, (**d**) 180, and (**e**) 270°. (**f**) Organized frequency-output result with changing string tension.

Figure 4 illustrates the frequency response changes induced by varying the tension of the string. The method for adjusting the string tension within the test setup is detailed in Figure 4a. Four representative tension levels were established by setting the winding angle of the lever to 0, 90, 180, and 270°. As the winding angle of the lever increases, both the tension and frequency of the string increase in accordance with Equation (1):

*f* = (*T*/*μ*)^1/2^/2*L*, (1)

where *f* represents the frequency of vibration, *L* is the length of the string, *T* denotes the tension of the string, and *μ* is the density of the string. This relationship elucidates how changes in the mechanical setup directly influence the vibrational characteristics of the string, subsequently affecting the frequency response detected by the sensor. This setup allows for precise control over the string’s vibrational properties, facilitating a detailed analysis of the sensor’s performance under varying tension conditions.

The frequency response to varying string tension is depicted in Figure 4b,e, where a low motor speed setting was utilized during the tests. As the winding angle increased from 0 to 180°, a gradual rise in peak frequency was observed, with the recorded frequencies being 51.89 Hz, 54.50 Hz, and 54.76 Hz in Figure 4b, Figure 4c, and Figure 4d, respectively. Upon further increasing the tension to a winding angle of 270°, the peak frequency slightly decreased to 54.60 Hz, indicating a cessation in frequency increase due to the material reaching its strain limit. Additionally, a significant 29.5% reduction in peak amplitude at the 270°-winding angle serves as further evidence of the string approaching its mechanical threshold. These data suggest that while increasing tension generally leads to higher frequencies, there is a critical point beyond which further tension does not yield additional frequency increases and may instead compromise the string’s structural integrity.

The organized results depicting the frequency responses for both output voltage and current are displayed in Figure 4f. In this figure, both electrical outputs demonstrate a consistent trend of increasing peak frequency, which eventually reaches a point of saturation. This behavior indicates a clear relationship between the applied mechanical tension and the electrical output, where increases in tension lead to heightened frequencies up to a threshold, beyond which no further increases are observed [41]. A detailed analysis of these frequency responses is further supported by the FFT spectra for the output current, presented in Figure S4. This figure shows the same trend in peak frequency and amplitude as those observed in the output voltage. These spectra provide a deeper insight into the specific frequencies at which the output current peaks, affirming the trends observed in the organized frequency results.

### 4.4. Integration of MCU and Visualization

**Figure 5:**
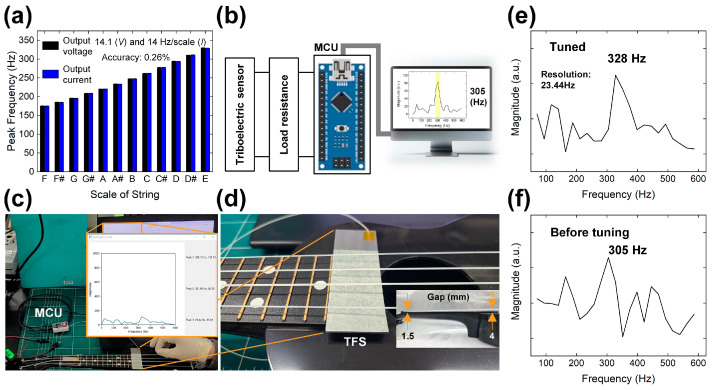
(**a**) Frequency sensing characteristic with changing scale of ukulele string. (**b**) Demonstration setup for frequency response sensing with TFS. Digital camera images of (**c**) frequency sensing system and (**d**) installed TFS under the string. Frequency sensing results with (**e**) accurate tuning and (**f**) inaccurate tuning.

The TFS was installed beneath the thickest string of a ukulele to assess its frequency response capabilities, as shown in Figure 5a. To verify the tuning accuracy, the results were cross-referenced with a commercial tuner attached to the ukulele’s head. As the tuning pegs were adjusted to increase the scale, the TFS accurately detected a corresponding increase in the peak frequency of both output voltage and current, ranging from 175 Hz to 330 Hz. By comparing the detected frequency values with musical notes confirmed using a commercial sensor (Yiwu Better Culture Co., Ltd. GTF52008, Jinhua, Zhejiang, China), the accuracy was found to be within 0.66% for output voltage and 0.69% for output current, respectively [42]. The FFT results, which detail the frequency distributions of the output voltage and current, are presented in Figures S5 and S6, respectively. These figures illustrate the precise manner in which the TFS responds to changes in string tension, reflecting an accurate increase in frequency as the strings are progressively tightened.

This setup demonstrates the ability of TFS to function effectively in a musical application, offering real-time, precise measurements that are essential for ensuring the ukulele is properly tuned. The integration of the MCU and subsequent data visualization via FFT not only confirms the sensor’s responsiveness but also enhances its practical utility in musical tuning applications. Following the initial measurements of basic electrical outputs and the analysis of frequency response with the TFS, the device was interfaced with an MCU of an Arduino Nano. This setup, illustrated in Figure 5b, enabled a comparison of the vibration frequency to a reference value. To ensure stable grounding, the TFS was connected to the analog sensing channels of the Arduino Nano via a parallel 1 GΩ resistor.

Once the output voltage signal was detected, it was transformed into a frequency spectrum using the Arduino code. This transformation utilized a sampling frequency of 3000 Hz and 128 samples, over a period of 0.5 s. The calculated resolution of the frequency response was 23.44 Hz, with a Nyquist frequency set at 1500 Hz. The resultant frequency spectrum was then transmitted to a Python program, which facilitated real-time visualization of the frequency spectrum and identification of the peak frequency value. This integration of Arduino IDE (version 2.1.0) and Python software (version 3.11.9) tools not only provided precise frequency measurements but also allowed for immediate graphical representation of the data, enhancing the analysis and usability of the TFS in practical applications.

The demonstration setup for sensing the tuning state of a ukulele and the installed configuration of the TFS is depicted in Figure 5c,d. In this arrangement, a glass substrate was meticulously positioned beneath the ukulele’s string, maintaining a precise gap distance of 3 mm. The gap distance between the string and the frets varied from 1.5 to 4 mm, as shown in the inset of Figure 5d. This strategic placement ensures optimal interaction between the string and the sensor without physical contact, thereby preventing any interference with the string’s natural vibrations. To initiate vibration and simulate typical playing conditions, the string of the ukulele was manually plucked using fingers. This method of activation not only reflects realistic usage scenarios but also allows for the accurate assessment of the responsiveness of TFS to changes in string vibration. The setup aims to investigate how effectively the TFS can monitor and analyze the frequency of vibrations, providing valuable insights into its potential applications in musical instrument tuning.

The demonstration of tuning state detection using the TFS is highlighted in Figure 5e and Video S1, which document the sensor accurately identifying the E scale tuning state of a ukulele. The peak frequency detected was 328 Hz, precisely matching the expected frequency for the E scale. This precise detection demonstrates the sensor’s capability to effectively monitor and confirm the correct tuning of string instruments.

Further demonstrating the sensor’s functionality, an example of an inaccurate tuning state is presented in Figure 5f and Video S2. Here, the pegs of the ukulele were deliberately loosened, resulting in the D scale tuning state, with a corresponding frequency of 305 Hz. This change illustrates the sensor’s responsiveness to alterations in string tension, enabling it to distinguish between different tuning states based on frequency changes.

This process confirms that the string instrument can be successfully tuned using the triboelectric vibration sensing system. By accurately detecting frequency variations corresponding to specific musical notes, the sensor proves to be a valuable tool for musicians and technicians alike, ensuring instruments are precisely tuned for optimal sound quality.

## 5. Conclusions

This study presented the development and application of a triboelectric frequency sensor (TFS) designed to detect the vibration frequency of strings. The schematic layout and operating principles of the sensor were thoroughly elucidated through illustrative figures. Optimization of the electrical output signals was achieved by adjusting the gap distance between the string and the contact dielectric material. The durability of the TFS was rigorously tested through 416,160 cycles, maintaining 99% of the initial output, and demonstrating its robustness and reliability. Variations in the speed of the motor and the tension of the string were methodically altered to evaluate the frequency response capabilities of the TFS. When applied to a ukulele, the scale tuning was accurately assessed by comparing the frequency results with those obtained from a commercial sensor. For the purpose of visualizing the frequency of string vibrations, a microcontroller unit and specific coding techniques were employed, linking directly to the TFS. The system’s efficacy was investigated through precise adjustments of the ukulele’s pegs, confirming the accurate tuning state. The proposed TFS-based system not only enhances the monitoring of string vibrations but also holds promise for broader applications, such as vibration detection in equipment within safety-critical fields. This technology paves the way for advanced monitoring solutions that are both effective and adaptable to various practical needs.

## Data Availability

Data available on request due to privacy restrictions.

## Supplementary Materials

The following supporting information can be downloaded at: https://www.mdpi.com/article/10.3390/mi15091079/s1, Figure S1: Operating principle with contact mode TFS; Figure S2: FEM results for non-contact TFS; Figure S3: FFT analysis of output current at different motor speeds; Figure S4: FFT analysis of output current with varied string tension; Figure S5: FFT analysis of output voltage across various ukulele tuning states; Figure S6: FFT analysis of output current across various ukulele tuning states; Video S1: Video of ukulele tuning within the accurate range; Video S2: Video of ukulele tuning below the accurate range.
