# Supplementary material for: Frequency Detection for String Instruments Using 1D-2D Non-Contact Mode Triboelectric Sensors
Source: Micromachines (Basel). 2024 Aug 26;15(9):1079. doi: 10.3390/mi15091079 (PMC11434310; doi:10.3390/mi15091079)
Supplement: Supplementary file 1 [file micromachines-15-01079-s001.zip › [20240826] (SM) String-TENG.pdf]

Supplementary Materials

Frequency Detection for String Instruments Using 1D-2D Non-Contact Mode Triboelectric Sensors

Inkyum Kim [1], Hyunwoo Cho [1] and Daewon Kim [2,3,*]

[1] Department of Electronics and Information Convergence Engineering, Institute for Wearable Convergence Electronics, Kyung Hee University, 1732 Deogyeong-daero, Giheung-gu, Yongin 17104, Republic of Korea
[2] Department of Electronic Engineering, Institute for Wearable Convergence Electronics, Kyung Hee University, 1732 Deogyeong-daero, Giheung-gu, Yongin 17104, Republic of Korea
[3] Center for BrainTechnology, Korea Institute of Science and Technology, 5, Hwarang-ro 14-gil, Seongbuk-gu, Seoul 02792, Republic of Korea
* Correspondence: daewon@khu.ac.kr (D.K.)

Table of contents

1. **Operating principle with contact-mode TFS**
2. **FEM results for non-contact TFS**
3. **FFT analysis of output current at different motor speeds**
4. **FFT analysis of output current with varied string tension**
5. **FFT analysis of output voltage across various ukulele tuning states**
6. **FFT analysis of output current across various ukulele tuning states**
7. **Video of ukulele tuning within the accurate range**
8. **Video of ukulele tuning below the accurate range**

1. Operating principle with contact-mode TFS

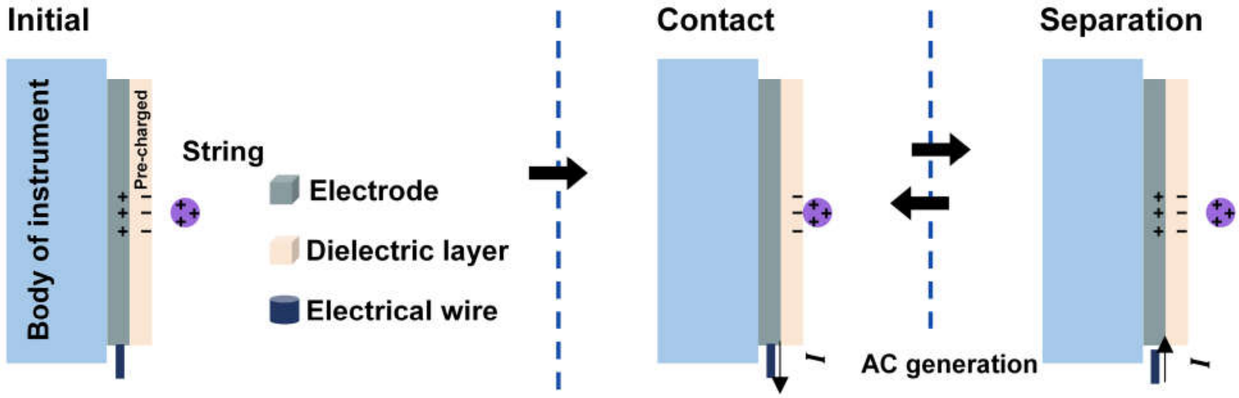

Figure S1. Operating principle with the contact between the string and the CDL.

2. FEM results for non-contact TFS

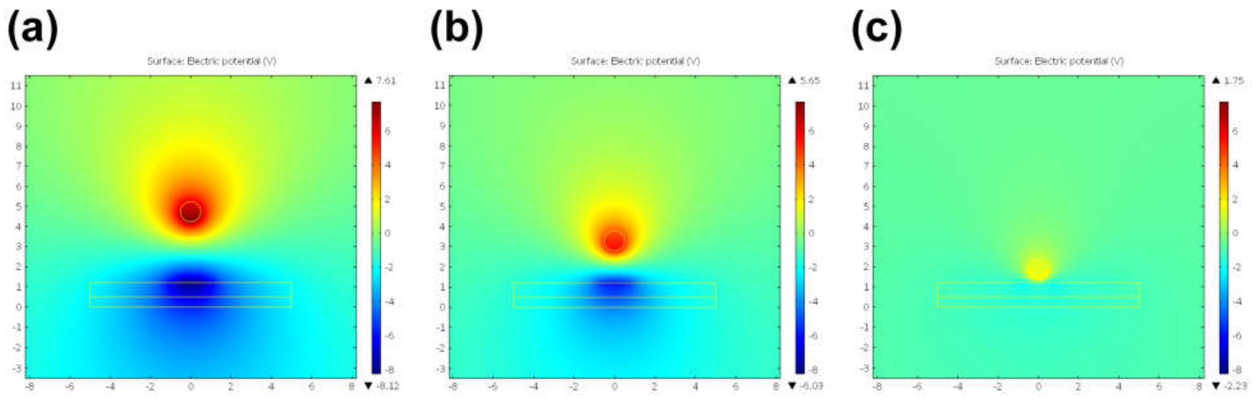

Figure S2. Potential distribution profiles of the non-contact TFS as analyzed using FEM: (a) 2.95 mm distance, (b) 1.5 mm distance, and (c) 0.05 mm distance.

3. FFT analysis of output current at different motor speeds

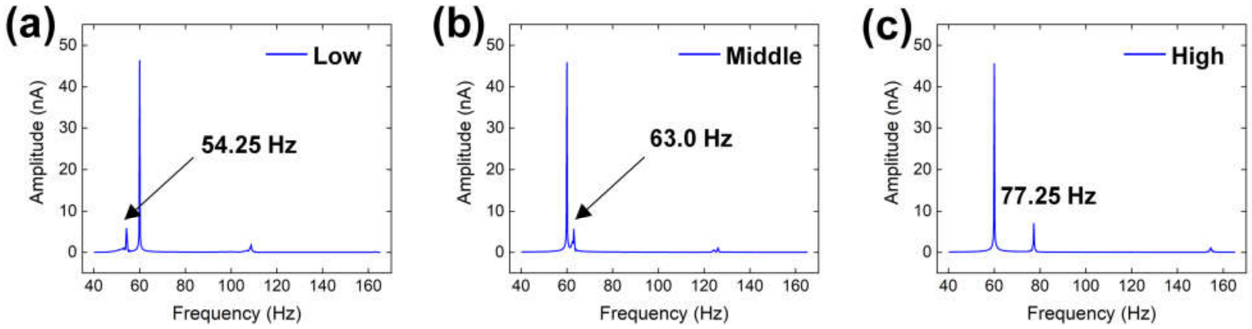

Figure S3. FFT spectra of output current with motor speeds: (a) low, (b) middle, and (c) high.

4. FFT analysis of output current with varied string tension

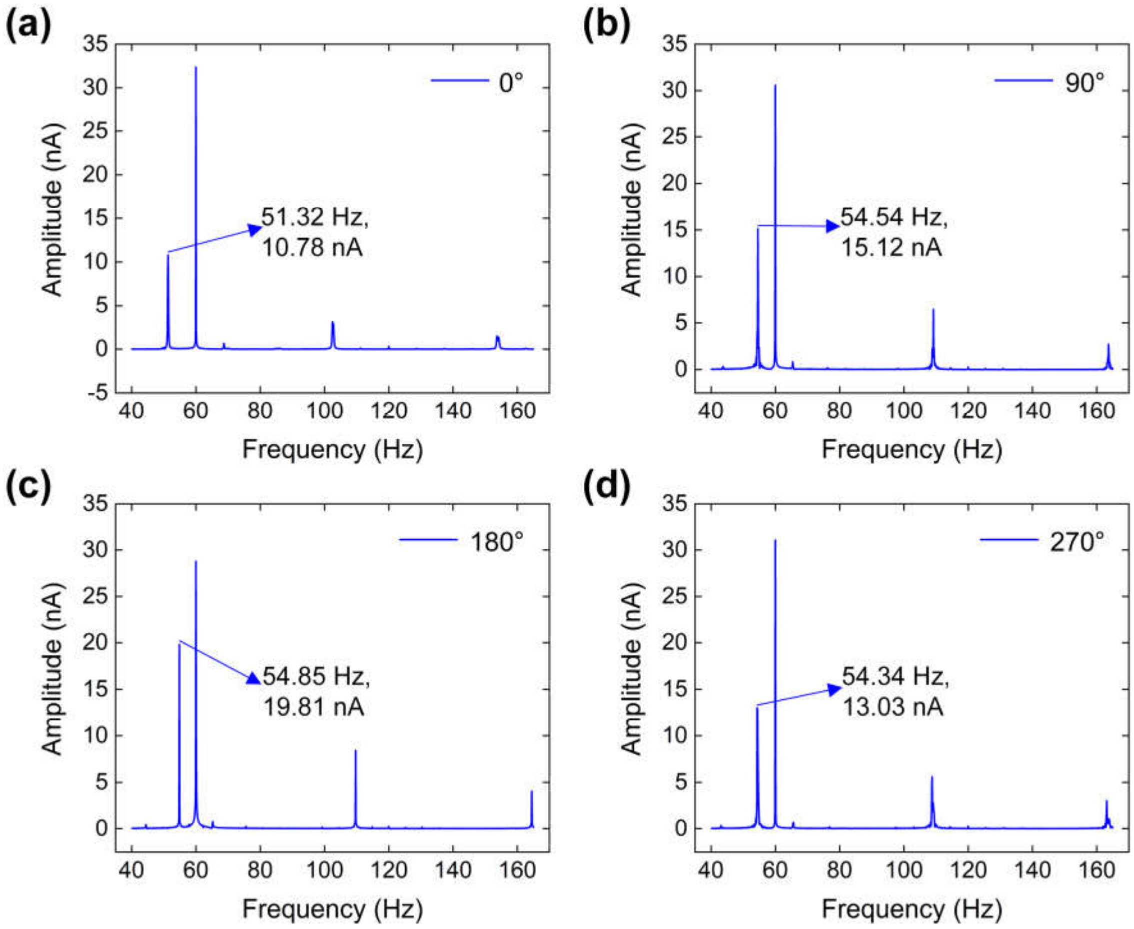

Figure S4. FFT spectra of output current with string winding angles: (a) 0, (b) 90, (c) 180, and (d) 270°.

5. FFT analysis of output voltage across various ukulele tuning states

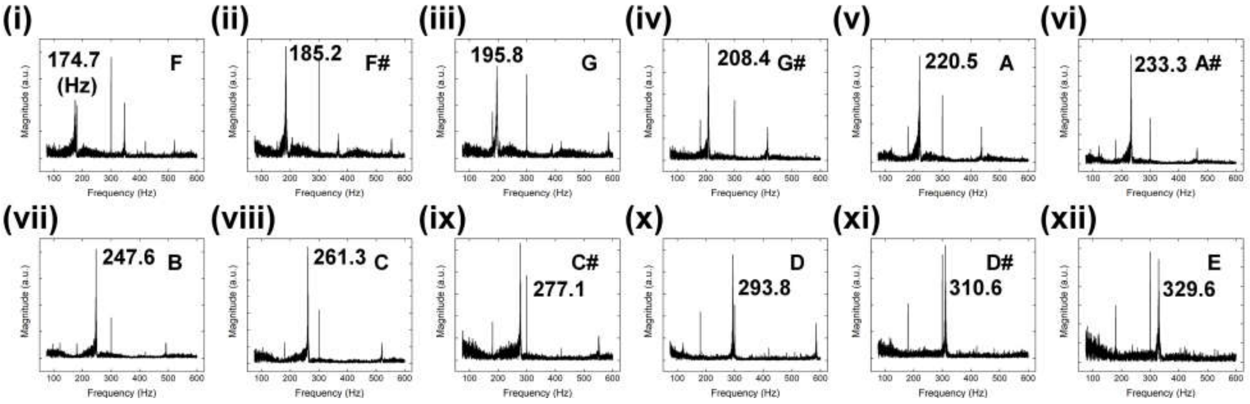

Figure S5. FFT spectra of output voltage with ukulele tuning states from F to E scale.

6. FFT analysis of output current across various ukulele tuning states

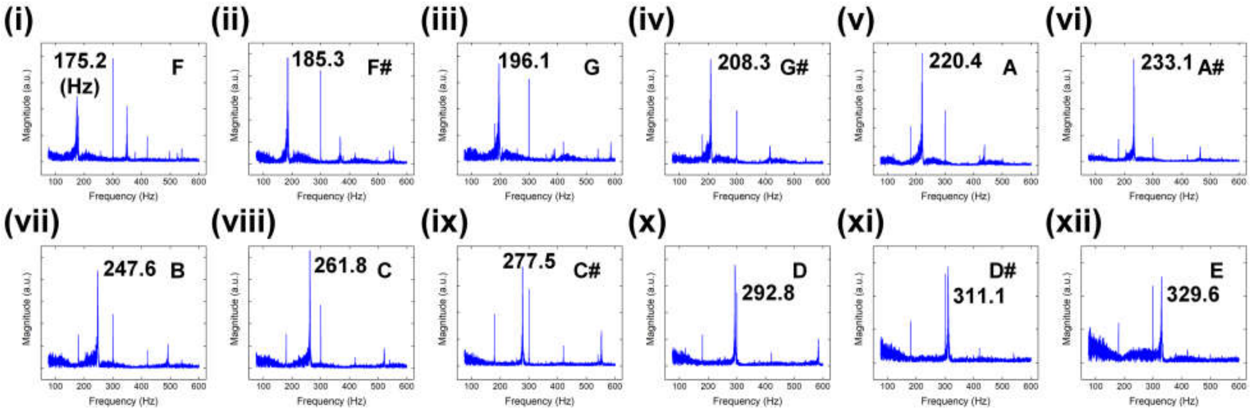

Figure S6. FFT spectra of output current with ukulele tuning states from F to E scale.

7. Video of ukulele tuning within the accurate range

Video S1. Demonstration of accurate tuning of the top second string of ukulele to the E scale.

8. Video of ukulele tuning below the accurate range

Video S2. Demonstration of inaccurate tuning of the top second string of ukulele to the D scale.
